# HEALTHCARE ACCESS AND SOCIAL ENGAGEMENT OF OLDER MINORITY ADULTS: EXPERIENCES NAVIGATING TRANSPORTATION BARRIERS

**DOI:** 10.1093/geroni/igz038.3297

**Published:** 2019-11-08

**Authors:** December Maxwell, Rebecca L Mauldin, Dennis Kao

**Affiliations:** 1 The University of Texas at Arlington, Arlington, Texas, United States; 2 Carleton University, School of Social Work, Ottawa, Ontario, Canada

## Abstract

Transportation is vital in the daily lives of older adults and provides access to health care services and health enhancing activities, such as social engagement. Disparities in mobility exist for older African American and Hispanic adults compared to non-Hispanic Whites, including higher likelihood of driving cessation at an earlier age and having a higher risk for reduced life space. This poster presents findings from a qualitative analysis of data from the Using Geo-Ethnography to Explore the Spatial Accessibility of Health Services for Aging Minorities Study (GeoSAS), a mixed methods study of older minority adults in Houston, TX. Using interpretive phenomenological analysis, the transcripts of semistructured interviews with 23 older adults (13 African American and 10 Hispanic; 17 female; mean age = 71.3 yrs, SD = 6.3 years) were analyzed to address the research question: What are the mobility experiences and perceptions of minority older adults regarding healthcare access and social engagement? Based on an ecological systems theoretical framework, we found reciprocal influences of (1) healthcare systems and transportation utilization and (2) participants’ health and well-being, mobility, and social engagement. Support from family members and financial capacity were critical for participants’ mobility. Implications of this research include educating health care providers about patients’ transportation experiences and barriers, optimizing social support to increase mobility, and addressing systematic disparities in transportation access to enhance health and well-being for older minority adults.

